# Measuring the benefits of the integration of health and social care: qualitative interviews with professional stakeholders and patient representatives

**DOI:** 10.1186/s12913-020-05374-4

**Published:** 2020-06-06

**Authors:** Helen Crocker, Laura Kelly, Jenny Harlock, Ray Fitzpatrick, Michele Peters

**Affiliations:** 1 Nuffield Department of Population Health, University of Oxford, Richard Doll Building, Old Road Campus, Headington, Oxford, OX3 7LF UK; 2 Harris Manchester College, Oxford, OX1 3TD UK; 3 Health Sciences, University of Warwick, Coventry, CV4 7AL UK

**Keywords:** Integrated care, Qualitative interviews, Outcome measures, Health and social care, Measurement

## Abstract

**Background:**

Integrated care has the potential to ease the increasing pressures faced by health and social care systems, however, challenges around measuring the benefits for providers, patients, and service users remain. This paper explores stakeholders’ views on the benefits of integrated care and approaches to measuring the integration of health and social care.

**Methods:**

Twenty-five semi-structured qualitative interviews were conducted with professional stakeholders (*n* = 19) and patient representatives (*n* = 6). Interviews focused on the benefits of integrated care and how it should be evaluated. Data was analysed using framework analysis.

**Results:**

Three overarching themes emerged from the data: (1) integrated care and its benefits, with stakeholders defining it primarily from the patient’s perspective; (2) potential measures for assessing the benefits of integration in terms of system effects, patient experiences, and patient outcomes; and (3) broader considerations around the assessment of integrated care, including the use of qualitative methods.

**Conclusions:**

There was consensus among stakeholders that patient experiences and outcomes are the best measures of integration, and that the main measures currently used to assess integration do not directly assess patient benefits. Validated health status measures are readily available, however, a substantial shift in practices is required before their use becomes commonplace.

## Background

Transforming the delivery of health and social care is needed to address the growing pressures due to funding constraints and increasing complexity of needs for services [1, 2]. In an attempt to address these issues, various programmes to integrate health services have been developed internationally [3, 4]. The World Health Organisation (WHO) recognises the importance of integrated care and recently developed a ‘*Framework on integrated people-centred health services*’ calling for fundamental change in the funding, management, and delivery of health services [5]. In England, many initiatives [6–9] have attempted to integrate the health and social care systems, with varying success, and most recently Integrated Care Systems (ICSs) are intended to cover the whole of England, as set out in the UK National Health Service (NHS) Long-Term Plan [10].

There are many different definitions and concepts of integrated care [11, 12]. A commonly referred to definition was developed by NHS England and National Voices, and takes a person-centred approach, “*I can plan my care with people who work together to understand me and my carer(s), allow me control, and bring together services to achieve the outcomes important to me*” [13]. While the integration of services can take many forms (e.g. horizontal or vertical), the focus of this paper is the integration of health and social care services. As an example, the Better Care Fund initiative pooled budgets to encourage local health and social care services to work more closely together to deliver ‘better, more joined-up services to older and disabled people, to keep them out of hospital and to avoid long hospital stays’ [14]. Initiatives funded through this scheme included multi-disciplinary teams, seven-day working, and single points of access.

Integrated care is difficult to achieve, especially in England where health and social care systems have a long standing institutional separation, with distinct funding and accountability arrangements. As such, it is important to be able to effectively evaluate the success of integrated care initiatives, and to achieve this, appropriate measures need to be available. However, questions have been raised about the suitability of existing measures, in particular, their breadth. Where instruments are available, there is a general lack of evidence to support their quality [15]. Furthermore, additional guidance around which measures and measurement instruments should be used is needed to aid comparisons of the success of strategies [16].

As integrated care arrangements continue to develop, there is a need to better understand their impact for patients, service users, and service providers. This study aimed to investigate how a diverse range of professional stakeholders and patient representatives viewed current approaches to assessing outcomes of the integration of health and social care. The findings are likely to be of international interest as countries attempt to evaluate the effectiveness of diverse integrated care arrangements and their impacts.

## Methods

Semi-structured interviews were conducted with stakeholders to explore the value of integrated care, identify aspects for evaluation to determine the success of integrated care, and identify appropriate measures for these aspects. Commonly, integrated care is pursued *for* patients and service users, without adequately acknowledging the potential contributions that they can make [17]. In an attempt to overcome this, patient representatives were included as active participants in this research, alongside professional stakeholders.

### Recruitment

Professional stakeholders were recruited through publically available information of national and regional NHS and social care representative bodies and stakeholder organisations, existing networks, and referral (i.e. snowballing) from those contacted or recruited using the above methods. Potential participants were selected based on their interest in and knowledge of integrated care services, and of policy/strategy and implementation in relation to integrated care. A purposive sampling strategy was adopted to recruit diverse respondents across the health and care sectors.

Patient representatives were recruited through the Quality and Outcomes of Person-Centred Care Policy Research Unit’s (QORU) Public Involvement and Implementation Group, a strategic group which oversees public involvement in QORU projects and includes members of the public.

Forty stakeholders and members of the Public Involvement and Implementation Group were sent an email invitation and participant information sheet about the study. Stakeholders were sent a reminder email where no response was received. We aimed to conduct 25 interviews across patient representatives, health and social care policy makers, system stakeholders and professionals. It was anticipated that this sample size would enable a range of perspectives regarding integrated care and its measurement to be captured thus providing detailed information about the evaluation of integrated care, the challenges and issues.

Ethics approval was granted by the University of Oxford’s Medical Sciences Interdivisional Research Ethics Committee (Reference: R59996/RE001). All participants provided written consent via a secure online consent form prior to taking part.

### Participants

Twenty-five people (19 professional stakeholders and 6 patient representatives) took part in a qualitative interview. Nineteen stakeholders responded to the email invitation and were interviewed. The remainder did not respond (*n* = 14), could not be reached (e.g. undeliverable email) (*n* = 6), or were too busy to participate (*n* = 1). Seven patient representatives contacted the research team expressing an interest in participating in the study, before recruitment was stopped due to sufficient numbers being reached. Six patient representatives were interviewed. The average duration of interviews was approximately 30 min. The roles and affiliations of professional stakeholders are summarised below (note that three stakeholders also held clinical roles):

- Two senior representatives of NHS England;
- Four stakeholders in lead roles within Clinical Commissioning Groups (bodies responsible for the purchase of local health care services);
- Three stakeholders in lead roles within other NHS bodies (e.g. Health and Care Partnerships);
- Four senior representatives of local authorities (bodies responsible for the provision of local public services, including social care). One of these representatives held a joint role with a Health and Care Partnership;
- One senior representative from the Local Government Association;
- Four senior representatives of health and/or care focussed charities;
- and a senior academic working in the field of integrated care.

### Interviews

A semi-structured interview guide (see Table 1) was developed following a review of relevant literature [18], and focused on the benefits of integrated care and how it should be evaluated. The guide was tested in two mock interviews (led by HC and LK), amended, and further refined after the first few interviews to allow broader exploration of available metrics for integrated care, and their selection. All interviews were conducted over the telephone between November 2018 and January 2019 (by HC, JH, or LK) and audio-recorded with the participants’ consent.

**Table 1:** Key questions asked in the semi-structured interviews

• Can you tell me about your current role and background? *(professional stakeholders)* / Can you tell me about you and your involvement in PPI (Patient and Public Involvement)? *(patient representatives)*	
• Can you describe what ‘integrated care’ means to you?	
• Can you tell me why you think integrated care is important?	
• If health and social care services are well-integrated, what benefits would you expect to see?	
• What aspects of integrated care do you think should be evaluated?	
• Do you know of any measures or tools that can be used to evaluate integrated care?	
• What factors do you consider when deciding which outcome indicators to use? *(professional stakeholders only)*	
• Do you think currently available measures are sufficient for evaluating integrated care, or are there areas where new metrics are needed? *(professional stakeholders only)*	
• What do you think is the best way to assess the impact of integrated care for patients and service users?	

### Analysis

Interviews were transcribed verbatim by a professional transcriber and checked against the audio-recording by a researcher (HC or LK), with corrections made as appropriate. All transcripts were anonymised.

A framework analysis was conducted in five stages: familiarization; identifying a thematic framework; indexing; charting; and mapping and interpretation [19, 20]. The thematic coding framework was developed following familiarisation with the data set, and broadly followed the interview guide. The framework was discussed and agreed upon by all authors. Next, the data was indexed using NVivo 12 software. Finally, a chart summarising the relevant data for each case against each qualitative theme was developed to aid interpretation.

Quotations from professional stakeholders are shown as “S” followed by an ID number. The following hierarchy of descriptors can be referred to when interpreting the results:

- Most or majority (≥75% of participants)
- Many or often (51–74% of participants)
- Some or several (26–50% of participants)
- A few or limited (≤25% of participants)

## Results

Three overarching themes arose from the data, and they are discussed in turn below. The first theme is concerned with how participants viewed integrated care and its benefits. The second theme focuses on potential measures for integrated care. The final theme explores some broader contextual and methodological considerations surrounding assessing benefits of integration initiatives.

### Integrated care and its benefits

When asked to define integrated care, the majority of stakeholders first and foremost defined it from the perspective of the patient or service user as an approach that coordinates services around the individual to fulfil their needs. Most went on to state that a key purpose of taking a person-centred approach to integrated care is to achieve better outcomes for patients and service users, for example, keeping people in their homes for longer, maintaining independence, reducing unnecessary admissions, improving health outcomes, and quality of life. In addition to improved outcomes, the potential for integrated care to provide patients with a better experience of care was also raised by stakeholders. Examples given of what a better patient experience may look like included: improved access to services, smoother transitions between services, increased involvement in decision-making, receiving the right care at the right time in the right place, and telling ‘their story’ less frequently.

Secondary to the person-focused definition offered above, some stakeholders provided a systems-focused perspective of integrated care, as an activity that coordinates or brings services together to provide ‘joined-up’ or ‘seamless’ care. Some outlined this vision in greater detail, specifying the services involved in integration (e.g. health, community, voluntary, and social care), as well as detailing the mechanisms by which integrated care can be achieved, for example, vertical or horizontal integration, and multi-disciplinary teams (MDTs). It is important to note that most stakeholders provided a systems-focused perspective in addition to the person-centred definitions given, outlining the structures and processes necessary in order to achieve better outcomes and experiences for patients. For example, one stakeholder spoke about how improvements to the structure of the health and care systems, specifically the integration of IT systems, could benefit patients:

“We don’t have a single joined up system. We don’t yet really have shared information systems which are incredibly important. And they're incredibly important enabler for integration. And so that’s what it's about for me – joining up information, making sure that care professionals have access to the right levels of information that they can use to make joint decisions that are with the patient as well, to improve the patient and service user outcomes.” (S10, Programme Lead, Clinical Commissioning Group)

Although structures and processes were discussed, the benefits of integrated care were generally coached in terms of the patient or service user. A few stakeholders further emphasised a person-centred approach by specifically stating that the end goal of integrated care is not the integration of structures, but rather the outcomes that can be achieved:

“It's [integrated care] about the experience and the improved outcomes that it brings to people, that is what it's all about; it's not about structures … it's about improving people's health and well-being, it's about closing health and inequalities, it's about keeping people well at home, giving them a better, more co-ordinated experience of care, about helping them to help themselves, about using, all of the assets in a place to support the community to live as happily and independently and safely as they can. And so that’s what integrated care is to us. It's not a thing in itself; it's the outcomes that it achieves.” (S9, Senior Advisor, Local Government Association)

The benefits of integrated care to service providers were also discussed, albeit less than the benefits to patients and service users, with two key areas of benefit raised. Firstly, many stated improved service efficiencies as a key benefit, achieved through more efficient pathways, reduced duplication of work (e.g. reduced number of assessments), reduced admissions, and an improved ability to make decisions, plan, and co-ordinate care. Secondly, the potential for integrated care to reduce costs was often discussed, although there were discrepancies between stakeholders regarding whether savings could be realised, with several acknowledging that this was not a given and that current evidence to support this was limited. In addition to these two key areas, a few stakeholders also highlighted the potential for integrated care to improve the experience and retention of staff.

Providing a definition of integrated care was more challenging for patient representatives. However, of those who were able, they provided similar albeit less detailed definitions than professional stakeholders, focused on bringing services together for the benefit of the patient:

“different organisations getting together … clinicians, carers, community people, researchers, research scientists, sharing knowledge and sharing anything that might be relevant to the patient that might improve the outcome to a patient” (Patient Representative 3)

Furthermore, patient representatives also provided similar descriptions of the benefits of integrated care, with a particular focus on streamlining processes to benefit the patient; for example, having one point of contact and being asked fewer times for the same information.

### Measures of integrated care

All professional stakeholders discussed how they thought integrated care should be evaluated, both in terms of the aspects to evaluate and how best to measure those aspects. Several professional stakeholders spoke about the difficulty of measuring the impact of integrated care, particularly with regards to the challenges faced when selecting appropriate measures, and assessing the benefits to patients and service users. Under current arrangements, with diverse measures employed across the system, a few stakeholders mentioned that it was impossible to draw general conclusions about the effectiveness of alternative models of integration. As one might expect, patient representatives discussed the measurement of integrated care in much less detail than professional stakeholders. While various system metrics were raised as possible measures of integrated care, patient representatives generally thought evaluation should focus on patients’ experiences of care, and any improvements in patient outcomes.

Three broadly distinct types of measure of the benefit of integration were discussed: system effects, patient experience and patient outcomes.

#### System effects

System metrics were commonly referred to by stakeholders when discussing measures of integrated care, of which, delayed transfers of care (DTOC), number of emergency admissions, and length of stay, all of which relate to hospital care, were among those most frequently mentioned. While there was great awareness among stakeholders of metrics used to assess system outcomes, and their value acknowledged, many felt that evaluations of integrated care focus too heavily on system effects. Concerns were raised that such an approach is too narrow and does not necessarily effectively evaluate the broader impact of integrated care and the benefits to patients. In particular, some regarded these metrics as ‘blunt instruments’ that do not capture the true impact and benefits of integrated care as defined by stakeholders, i.e. whether system changes are improving patient experiences and outcomes:

“The outcomes often associated with integrated care are hardcore metrics like reductions of emergency admissions, unplanned admissions, length of stay and that sort of thing … these are the metrics that they use to measure integrated care against, don’t actually demonstrate an effective connection, and that’s because those metrics in themselves aren't particularly sensitive to what is the aspiration of integrated care.” (S11, Senior Academic, University)

Vast awareness and use of system metrics is, in part, likely explained by the requirement of regulators to collect data on certain system outcomes, for example, Delayed Transfers of Care (DTOC). An Assistant Director at a local authority questioned this focus:

“The sign of a good performing system is that you have low levels of DTOC … yes, it's nice to have a low level of DTOC, but that still means that the person's been in hospital. They could have been in hospital for three or four weeks. Is that necessarily a good experience for them? … Sometimes we latch onto a particular measurement and then say, "Oh yeah, your system's performing well because you’ve got a really low DTOC." Well, not if you’ve got really high non-elective admissions and really high readmissions and really high residential admissions … that tells me that the system's not operating as well as it could.” (S4, Assistant Director, Local Authority)

#### Patient experiences

The majority of participants spoke about the need to measure patients’ experiences, with a few specifically extending this to include the experiences of carers and / or family members. Furthermore, several thought it also important to measure the experiences of the workforce.

Patient representatives frequently referred to experiences of care, talking about their own experiences from a patient or carer perspective, as well as referring to the experiences of others (e.g. family, friends and neighbours). One patient representative described the experience of their neighbour:

“She suffered a mental health breakdown with two young children, and the [city] social services department took six months to assess her. And even then they didn’t complete the assessment because the lady was homeless. And they moved her to temporary accommodation into the neighbouring borough of [location], where they had to start the whole process again because they said they'd lost the papers … So, there's no communication between one department and another department.” (Patient Representative 1)

This focus reinforces the stakeholders’ views of the importance of measuring patients’ experiences of care.

Some difficulties around measuring patients’ experiences were raised by stakeholders, for example, often patients and service users provide a positive report of their care despite negative aspects occurring, which often only come to light during subsequent discussions with the patient.

#### Patient outcomes

In general, stakeholders discussed patient-related outcomes in broader terms than they had discussed system effects. For example, some stakeholders advocated the importance of patient outcomes but generally without specificity or detail. This may be an effect of system outcomes being measured more frequently, and therefore, system metrics being better known to stakeholders than patient outcome measures. This was not true of all, with a few participants able to discuss dimensions to be measured (e.g. wellbeing, functionality, and pain), and tools that could be used to elicit such data, in much greater detail.

Several stakeholders called for a wider “*basket of measures*” to broaden measurement beyond system effects when evaluating the success of integrated care programmes. A few felt that measuring patient outcomes should be central to evaluations, for example, a Senior Associate of a charity stressed that “fundamentally, the best judge of a service or a package of services, is from the perspective of the people experiencing care” (S1, Senior Associate, Charity).

Overall, very few specific health indicators were referred to, with life expectancy, functionality, and dental decay among those mentioned. The majority of discussion was about patient- or carer- reported outcomes. Quality of life, well-being, or specific aspects of quality of life, such as pain, anxiety, and depression were raised by the majority of stakeholders as aspects of integrated care that should be evaluated. The value of measuring aspects such as these was highlighted in an example shared by a Senior Manager, who described an instance where a GP visited an elderly man with multiple conditions nearing end of life:

“And the GP thought, 'Well what can I do for this guy?' and he... so he just asked him. He said, "I'd really like my beard trimmed." So, he kind of got a barber to come round and trim his beard and he said it kind of transformed this guy's kind of, you know, you know, made him feel so much better. And his blood pressure was lower and things like that. We don’t... but we don’t kind of pick that up. I think all you'd pick up was the fact that whether the GP went there and how long he was there for, or she was there for, you know.” (S6, Senior Manager, County Council)

However, a few held some reservations about collecting such data due to its subjective nature, and problems with measurement (e.g. comparability):

“I might say my quality of life's really high and I enjoy my life; I could live in absolute squalor. And other people would say my quality of life is very poor. So, I think it's more difficult around quality of life … I think it is right that we ask the question, but I think it's one of those ones that you need to think about that is subjective.” (S4, Assistant Director, County Council)

#### Patient-reported measurement instruments

When asked about available patient-reported measures for assessing integrated care, broadly speaking, stakeholders working at a national level (e.g. national charities) and in academia were far more familiar with specific measures than those working at a local level (e.g. County Councils and Clinical Commissioning Groups). Measures named, included, the Patient-Centred Coordinated Care Experience Questionnaire (P3CEQ), the Friends and Family Test, the Patient Activation Measure (PAM-13), the Warwick-Edinburgh Mental Well-being Scale (WEMWBS), and the EuroQol 5 Dimensions (EQ-5D). None of the measures named were strongly advocated by the professional stakeholders. Although patient representatives did not specifically name any patient-reported measurement instruments, many had previously seen or completed patient-reported outcome or experience questionnaires as part of their care. One patient representative described their experience:

“I have endless review questionnaires following interventions about, you know, pain scores and all that sort of stuff. Can we do this, can we do that more, you know, was it better, was it worse, was it just the same. But no, I personally, have a bit of trouble with those as a patient because ticking boxes and being a human being don’t seem very compatible to me, but I do understand that it's the way we measure things. And I know you get something from it which translates into something which will help me.” (Patient Representative 4)

### Contextual considerations

In addition to discussing potential measures for integrated care, stakeholders also raised some additional factors for consideration when evaluating integrated care programmes. Both professional stakeholders and patient representatives raised queries around the purpose of data collection in health and care settings. One patient representative discussed the lack of transparency with patients regarding the purpose for which data is collected, referring to “*endless surveys*” that go “*into a dark hole and you assume that nobody ever reads”* (Patient Representative 4). Several professional stakeholders called for greater clarity around why data is being collected and how that data will be used:

“The other thing that worries me is are we using that data? … it's very fashionable to talk about, you know, people's mental health and all this sort of stuff, so people feel that they ought to collect it, but people should only be collecting it if they're going to use it, you know. If, you should only be asking these people these questions if they’ve got a plan for that data. If they just want to kind of want to put it on a database somewhere and forget about it then it’s unethical.” (S6, Senior Manager, Council)

A further issue, raised by a few stakeholders, was around the tensions between collecting data for use at a local or national level. While stakeholders recognised the need to collect data at a national level to allow comparisons to be drawn between providers, there were concerns that national level data lacked local relevance, and necessitated a loss of local data ownership. An Assistant Director at a Council questioned the value of nationally imposed metrics:

“We are supposed to work in an integrated way and cooperate, but I think the culture that underpins that has been one of intimidation from the top. An insistence of further solutions by certain providers being delivered, and whether you’ve got a top driven set of metrics that have come from NHS England and don’t reflect the reality of local government funding. Those metrics become either as just a stick to beat people with. And it doesn’t help the integration of human beings working together on the ground; it gets in the way.” (S15, Assistant Director, Council)

#### Qualitative methods

Qualitative methods of evaluating integrated care were increasingly being used or advocated by many stakeholders in order to overcome difficulties they had experienced with quantitative evaluation of integrated care initiatives. A senior academic discussed their qualitative approach to evaluation:

“We get people to define what is it you wanted to get out of it and did integrated care actually help you? So, it's impossible to plonk it on a politician's desk and say, 'You know, with this integrated care initiative, X number of outcomes were achieved,' because it's not like that. Integrated care is much more evaluation of a process. And individually, did the processes actually facilitate some kind of improvement?” (S11, Senior Academic, University)

Stakeholders often discussed the use of qualitative methods to capture data about the benefits of integrated care from the patients’ perspective. Some advocate these methods as a way of hearing the stories of patients. Despite discussing the benefits of a qualitative approach, it was acknowledged that there is still a need for good metrics with which to evaluate and report on integrated care.

“Asking the person at the end of it how integrated their experience has been and then acting on what they tell us. It's experiential, it's qualitative and it's not going to be easily captured and quantified and reported upon.” (S15, Assistant Director, Council)

It has to be noted that while many stakeholders referred to “*qualitative*” approaches, it was evident that a few were in fact referring to patient experience surveys rather than qualitative methodological approaches, such as interviews and focus groups.

## Discussion

In this diverse sample of professional stakeholders and patient representatives with a range of interests in the process of integrating health and social care, there was striking optimism that increased integration would eventually yield substantial benefits to patients and users of services. There was consensus that currently health and social care services are a very long way from being integrated and that the main measures used to assess progress, such as delayed transfer of care, while important, do not directly address patient benefits.

When invited to consider how benefits of integration should be assessed, participants commonly referred to broadly expressed dimensions such as well-being and health-related quality of life. However such constructs were generally seen as difficult to operationalise and little specific evidence was cited of integration being assessed against outcomes such as well-being. Reference was also made by some participants to the potential value of capturing patients’ experiences of services, i.e. how services were experienced in terms of being joined up and coordinated. For some, such responses to integrated care would best be captured by qualitative and narrative methods.

Although there was broad agreement that progress in integration would be best captured through the measurement of experiences and outcomes (e.g. dimensions such as well-being), no single measure was widely cited. However, individuals were able to cite specific measures such as the Patient Activation Measure or EQ-5D as potentially relevant. Furthermore, stakeholders generally discussed the applicability of measures in broader terms rather than considering their relevance to specific groups.

Related to the lack of agreement about specific measures of outcomes of integration was widespread uncertainty as to where responsibility for outcomes assessment was best located, at local or national level. This uncertainty reinforced the sense that no shared metric could easily emerge that would inform the system as a whole of benefits of initiatives to develop integrated services.

The development of measures of health status, often referred to as patient-reported outcome measures, has been hailed as the most important scientific break-through in fifty years, permitting the evaluation of services by means of broad measures of health as viewed and valued by patients and the public [21]. There are now readily available validated measures of health intended to capture the benefits of system innovations in health and social care [22, 23]. There are, in addition, attempts to define sets of indicators to assess and monitor integrated care [24], and logic models that delineate relationships between systems and outcomes [25]. It is clear that there still remains a substantial gulf between health services research and the world of everyday service providers, managers and commissioners, which is holding back the use of evaluative methods to inform innovation [26]. One aspect of the gulf is limited awareness of potentially relevant patient-reported outcome measures and their application.

One way in which this gulf could be narrowed is through the development and promotion of a core measurement set for integrated care, i.e. a standardised set of measures that represent the minimum group of processes, structures, and outcomes that should be collected and reported for all integrated care initiatives. This type of approach follows that set out by initiatives such as the International Consortium for Health Outcomes Measurement (ICHOM), which focuses on the development of Standard Sets for use by health care providers [27], or the Core Outcome Measures in Effectiveness Trials (COMET) initiative, which aims to standardise measurement across clinical trials for a specific area of health or health condition [28]. At the heart of this type of approach is the identification and agreement of what matters most to patients and service users in terms of outcomes from services, together with the identification and agreement about best available specific measures of the most important outcomes.

A potential weakness of these conclusions is the sample size of respondents recruited to the study. An enormous array of professional, managerial and patient groups have an interest in the broad issue of integration of health and social care and the current study cannot claim to have fully sampled this diversity of views. The recruitment of professional participants with in-depth knowledge of integrated care led to a sample of professionals in more senior positions. However, further work would benefit from the inclusion of professionals of varying levels of seniority to capture a wider range of views. Patient representatives were less familiar with the term ‘integrated care’ and its definition, with some finding it difficult to answer some of the questions as a result. However, it is essential to include patient representatives, and further work is needed to explore how best to overcome terminology and conceptual barriers to participation. Interviews were also pragmatic and restricted in time to accommodate respondents’ time constraints.

This study makes an important contribution to understanding professional and lay stakeholders’ views on capturing the benefits of integrated care, but further work is needed to better understand the challenges of measuring health outcomes and patient, informal carers, and staff experiences as part of integrated care evaluations. Patients and informal carers should be central to the development or selection of measures and indicators to ensure that they truly reflect any differences that integrated care has made.

## Conclusions

The challenges of measuring integrated care are well documented. In this paper, we sought the views of professional stakeholders and patient representatives about how best to measure the integration of health and social care, and the benefits of integrated care approaches. Stakeholders primarily defined integrated care and its benefits from the patients’ perspective, however, there was consensus among stakeholders that there is not yet enough focus on patient benefit. There is potential for this to be overcome as validated patient-reported measures of health are available. However, there is currently limited awareness of measures of key constructs such as wellbeing among stakeholders, and therefore considerable scope for developing consensus on optimal measures. The study provides clear evidence that the prospect of directly assessing patient benefits of the integration of health and social care services is feasible, although still quite distant.

## Data Availability

Due to ethical concerns, the interview transcripts cannot be made openly available.

## Abbreviations

COMET: Core outcome measures in effectiveness trials
DTOC: Delayed transfers of care
EQ-5D: EuroQol 5 dimensions
ICHOM: International consortium for health outcomes measurement
ICS: Integrated care systems
MDT: Multi-disciplinary team
NHS: National health service
P3CEQ: Patient-centred coordinated care experience questionnaire
PAM-13: Patient activation measure
QORU: Quality and outcomes of person-centred care policy research unit
WEMWBS: Warwick-Edinburgh mental well-being scale
WHO: World health organisation
